# Supplementary material for: Hypoxia Regulates Endogenous Double-Stranded RNA Production via Reduced Mitochondrial DNA Transcription
Source: Front Oncol. 2021 Nov 24;11:779739. doi: 10.3389/fonc.2021.779739 (PMC8651540; doi:10.3389/fonc.2021.779739)
Supplement: Supplementary file 1 [file DataSheet_1.pdf]

Supplementary Figure 1

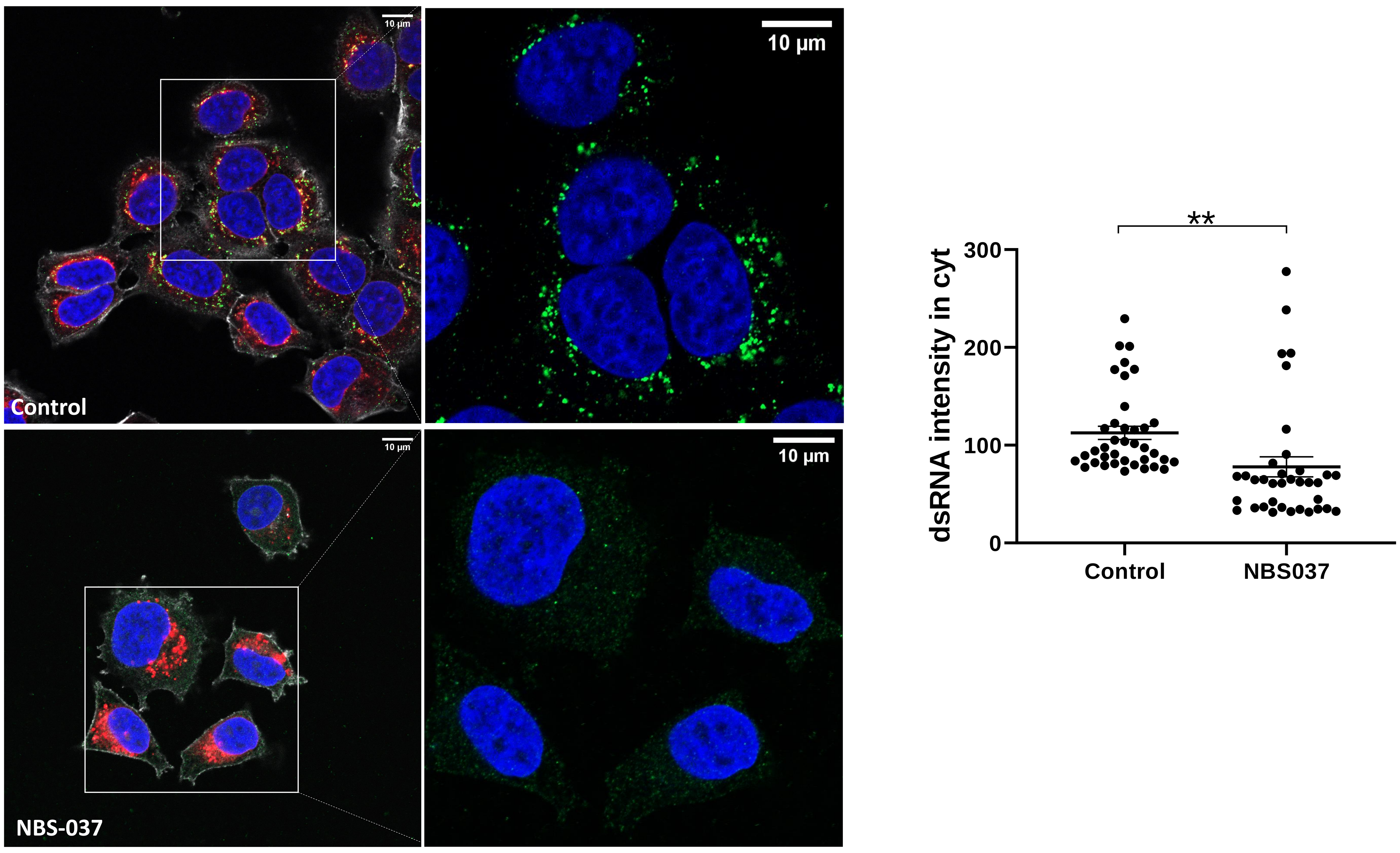

Supplementary Figure 1. NBS-037 treatment reduced dsRNA levels in MCF7 cells. dsRNA was stained using J2 antibody in MCF7 cells cultured in normoxia and treated with a control vehicle (DMSO) (n=38 cells) or 1µM NBS-037 (n=36 cells) for 48h from 3 independent replicates. Data is shown as mean±SEM. * p<0.05, ** p<0.01, *** p<0.001. Green: J2 antibody staining, blue: DAPI, and red: MitoTracker staining. Scale bars correspond to 10µm.

Supplementary Figure 2

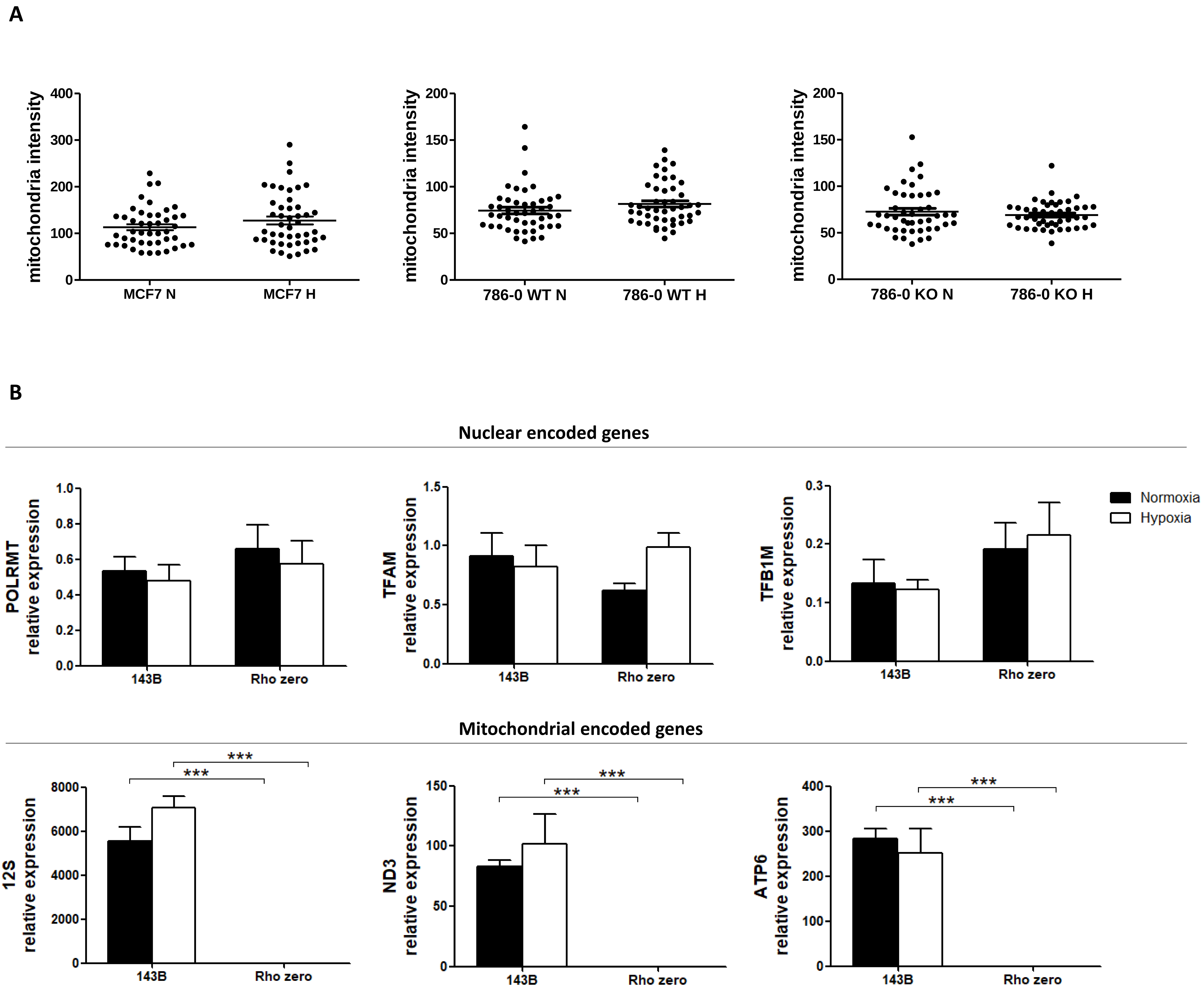

Supplementary Figure 2. Hypoxia does not affect mitochondrial content in MCF7 or 786-0 cells and expression of mitochondrial genes in 143B parental cells. (A) Mitochondria staining with MitoTracker was quantified in MCF7, 786-0 WT and 786-0 KO cells cultured in normoxia or 0.1% hypoxia for 48h (n=3). (B) RNA expression of mitochondrial encoded genes (*12S, ND3, ATP6*) or nuclear encoded genes (*POLRMT, TFAM, TFB1M*) involved in mitochondrial function was evaluated by qPCR in 143B parental and Rho Zero cells cultured in normoxia or 0.1% hypoxia (n=3). Number of replicates indicate biological replicates and data is shown as mean±SEM. * $p<0.05$, ** $p<0.01$, *** $p<0.001$

Supplementary Figure 3

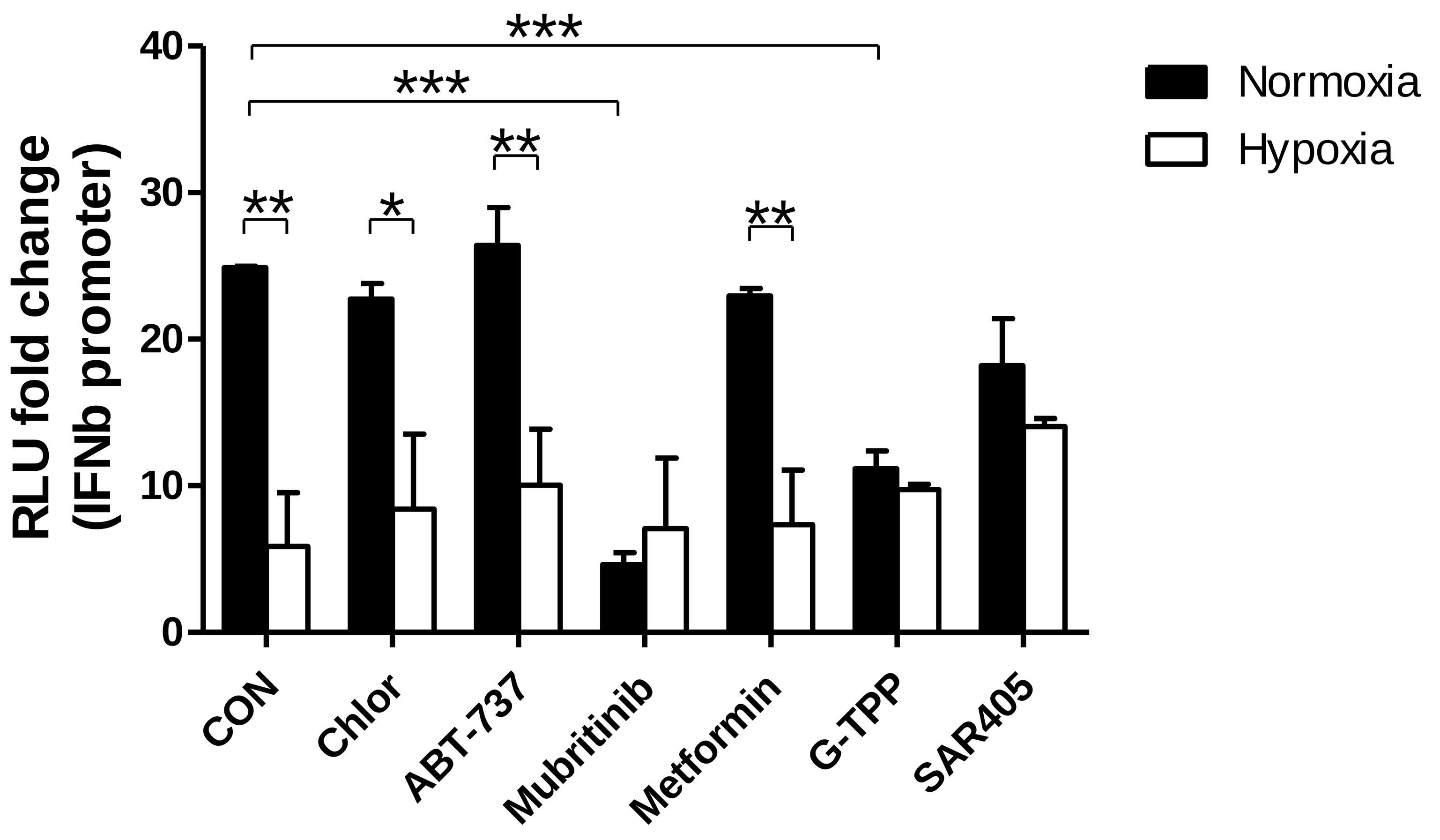

Supplementary Figure 3. Hypoxia has a major role over the effect of different mitochondrial-targeting drugs on *IFNβ* promoter activation. *IFNβ* promoter stimulation was evaluated in MCF7 cells cultured in normoxia or 0.1% hypoxia for 48h and treated with different drugs targeting different mitochondrial processes (n=3). Number of replicates indicate biological replicates and data is shown as mean±SEM. * $p<0.05$, ** $p<0.01$, *** $p<0.001$
