# Supplementary material for: Hypoxia Regulates Endogenous Double-Stranded RNA Production via Reduced Mitochondrial DNA Transcription
Source: Front Oncol. 2021 Nov 24;11:779739. doi: 10.3389/fonc.2021.779739 (PMC8651540; doi:10.3389/fonc.2021.779739)
Supplement: Supplementary file 2 [file DataSheet_2.pdf]

**Supplementary table 1. siRNA sequences.**

| siRNA | Sequence (5'-3') |
|---|---|
| siCON | ACGACACGCAGGUCGUCAUTT* |
| siBNIP3 | UCGCAGACACCACAAGAUA |
| | GAACUGCACUUCAGCAAUA |
| | GGAAAGAAGUUGAAAGCAU |
| | ACACGAGCGUCAUGAAGAA |

**Supplementary table 2. Antibodies.**

| Protein | Antibody | Company | Dilution |
|---|---|---|---|
| β-actin | A3854 | Sigma | 1:50000 WB |
| HIF1a | 610958 | BD Transduction Lab | 1:1000 WB |
| HIF2a | NB100-122 | Novus Biologicals | 1:500 WB |
| SUV3 | A303-055A | Bethyl Laboratories | 1:1000 WB |
| PNPT1 | Ab96176 | Abcam | 1:1000 WB |
| J2 | 10010500 | Scions | 1:200 IF |
| PKR | sc-6282 | Insight Biotechnology Ltd | 1:50 IF, 1:500 WB |
| phospho PKR (T446) | ab32036 | Abcam | 1:1000 WB |

**Supplementary table 3. Primers.**

| Primers | Sequence (5'-3') |
|---|---|
| HPRT1_F | TGACACTGGCAAAACAATGCA |
| HPRT1_R | GGTCCTTTTCACCAGCAAGCT |
| 12S_F | ATATACCGCCATCTTCAGCA |
| 12S_R | CTAAATCCACCTTCGACCCT |
| ATP6_F | GGACTCCTGCCTCACTCATT |
| ATP6_R | AAGTGGGCTAGGGCATTTTT |
| TFAM_F | GCTAAGGGTGATTCACCGCA |
| TFAM_R | ATCCTTTCGTCCAACTTCAATC |
| POLRMT_F | AGGTCAAGCAAATAGGAGGTG |
| POLRMT_R | CAGCGAGTGGATGAAGTTGG |
| CYTB_F | CGCATGATGAAACTTCGGCT |
| CYTB_R | ATTTGGAGGATCAGGCAGGC |
| ND3_F | GGCTTCGACCCTATATCCCC |
| ND3_R | TAGGGCTCATGGTAGGGGTA |
| TFB1M_F | TGCTTGCCGCGTATCATG |
| TFB1M_R | CGGAGGGAGACGGCAAGT |
| MRPL1_F | TTTACAGAGAATGCATCAGAGG |
| MRPL1_R | AGGCATTATTTCTGGAACAGC |
| MRPL11_F | GAGGCGTTTCCATCAACCAG |
| MRPL11_R | CACCTCTTTCCCTGTTTGCC |
| MRPL13_F | TACTAGGAGAAGGACGTACGG |
| MRPL13_R | CACAGTCACTCAGTGCATGG |
| MRPL21_F | GGAAATGAACTAGACCTTGCGT |
| MRPL21_R | AAGATCCTTTCCGAGGAGTG |
| MRPS34_F | GTGGACTACGAGACCTTGAC |
| MRPS34_R | AAAGAGGCGTCTTTGAAGGTC |
| MRPS23_F | GCTCCCATCCAAGACATCTG |
| MRPS23_R | TACTTCTCCACAAACCGTTGAC |

| | |
|---|---|
| DDX58_F | CAAGCCTTCCAGGATTATATCCG |
| DDX58_R | AGTCCAGAATAACCTGCATGGT |
| ADAR-p150_F | CTTCCAGTGCGGAGTAGCG |
| ADAR-p150_R | GTGACGGTGTCTGCTTTCCA |
| Mx1_F | GTTACCAGGACTACGAGATTGAG |
| Mx1_R | GATGAGTGTCTTGATCTTATACCC |
| IFIT1_F | TACCTGGACAAGGTGGAGAA |
| IFIT1_R | GTGAGGACATGTTGGCTAGA |
| IFIT2_F | TGTGCAACCTACTGGCCTAT |
| IFIT2_R | TTGCCAGTCCAGAGGTGAAT |
| ISG15_F | GCGAACTCATCTTTGCCAGTA |
| ISG15_R | CCAGCATCTTCACCGTCAG |
| PKR_F | ATCTGACTACCTGTCCTCTG |
| PKR_R | GAGACCATTCATAAGCAACGA |
